# Observations on Cancrum Oris, or Sloughing Sore Mouth of Children

**Published:** 1839-08-31

**Authors:** W. T. Webb

**Affiliations:** Alabama, Senior Resident Physician to the Philadelphia Hospital, Blockley


### MEDICAL EXAMINER.

DEVOTED TO MEDICINE, SURGERY, AND THE COLLATERAL SCIENCES.

No. 35.] PHILADELPHIA, SATURDAY, AUGUST 31, 1839. [Vol. II.

*Observations on Cancrum Oris, or Sloughing Sore Mouth of Children.* By W. T. Webb, M. D., of Alabama, Senior Resident Physician to the Philadelphia Hospital, Blockley.

This disease came under my observation in 1838, at which time I was resident physician in the Children's Asylum. It followed a very severe epidemic of rubeola which prevailed in that institution, commencing in May, and terminating in August. This disease, which is sometimes (and with more propriety) termed gangrenous sore mouth, is seldom witnessed; when it does occur, it is most frequently seen in charitable institutions, where a large number of children are congregated together. We do not find it, however, entirely confined to such asylums. Dr. B. H. Coates, in vol. ii. of the North American Medical and Surgical Journal, mentions that this disease at one time prevailed in New Jersey. It was there considered as a sequela of intermittent and remittent fevers. It seldom occurred except in marshy districts, and among the children of the poor. A few cases have been treated by some of the physicians of Philadelphia. We witnessed twenty-three cases in the asylum. Most of the children which were the subjects of gangrenous sore mouth, had been previously attacked with rubeola.

At Paris the disease is quite common at the Children's Hospital, and there generally occurs after an attack of measles; it is by no means confined to this disease, but may be said, in general terms, to attack any children of enfeebled constitutions; although it is certainly true, that measles exercises a peculiar influence in favouring its development. Children are at times brought into the hospital from the city of Paris, but the great majority of cases undoubtedly originate within the building.

*Description of the disease.*—This disease generally commences at the edges of the gums opposite the incisors of the inferior maxillary bone. The first change which we could perceive in the gums, was a whiteness much resembling the appearance which healthy gums would assume immediately after a very superficial application of the nitrate of silver in substance. They also became quite spongy; and, by closely inspecting the gums, we would find them separated from the teeth. At this stage it now closely resembled ptyalism; indeed, so close is the resemblance, that it is almost impossible to distinguish the one from the other; but in this disease there was not that great increased secretion of saliva which almost always accompanies salivation. Dr. Coates tells us, that Dr. Parrish for several years in his private lectures noticed a stage of this disease, under the name of "*a disease resembling the effects of mercury.*"

If the disease be not arrested, ulceration commences and extends along the gums, until the whole jaw becomes more or less incurably affected. Not unfrequently, both the superior and inferior maxillary bones become involved. In two cases, the ulceration commenced simultaneously in both. As the disease advanced, the cheeks or lips began to swell, until they became tense and hard, white and glossy. "The great distention of the skin seems to empty the cutaneous vessels, giving to the part a smooth, polished appearance, very much resembling the effects of severe salivation.

"The gums are now gradually removed by ulceration,—the teeth become loose, and if not removed, fall out spontaneously, denuded of their periosteal covering. By some it is supposed, that the periosteum is the part which is the most peculiarly liable to injury and death, and that it is in this membrane that the disease not unfrequently begins; but this is by no means certain. The gums and inside of the cheeks soon assume a gangrenous appearance, the breath also becomes extremely fœtid. After reaching this stage, the exterior of the cheek, or the part where tumefaction existed, presents a dark purple colour; and in many cases vesicles form, from one-fourth to one inch in diameter, filled with a dark coloured fluid, which soon burst and discharge, and in a very short time are followed by sloughs." As soon as the external gangrene has reached the level of the bony socket, and frequently at an earlier period, the adjacent portions of the alveoli are found deprived of life, forming a necrosis. The death of the periosteum in the socket, at least that of the fang of the tooth, precedes by some interval of time, that of any portion of the bone itself.

The discharge from the gangrenous surface was very acrid, producing excoriations of the cheeks, lips, tongue, or any other part with which it came in contact.

In the early stages of gangrenous sore mouth, it seems to have but little effect on the general health of the patient. The pulse remains natural, skin cool, appetite good, and in many cases but little pain was complained of, except in the application of the remedies, which generally were of a highly stimulating character. In the last stages of this disease, it was attended with much constitutional disturbance. The pulse became quick and small, skin hot and dry, appetite much impaired, and, in some cases, complete anorexia. The patient frequently became extremely emaciated, colliquative diarrhœa now set in, and death closed the scene. We cannot better illustrate this disease, than by laying before our readers the notes of a very interesting case which Dr. Halson, of Virginia, my successor as resident physician in the asylum, has kindly furnished me.

*Case.*—John Smith, æt. six years, was convalescent from pneumonia. On the 1st of March, a white border was discovered around the gums, opposite the incisors of the inferior maxillary bone. The gums were spongy, and somewhat separated from the teeth; they are not disposed to hæmorrhage; no increase of the salivary secretion; appetite good; pulse natural; bowels regular; sleeps well.

℞. Tr. Kreosote ʒii.

Aquæ ℥vi. M.

Apply to gums three times a day.

*March 3d.*—But little change since last note, except that the edges of the gums are slightly ulcerated. Continue treatment.

*5th.*—Ulceration continues to increase; it has extended down towards the roots of the incisors. Appetite remains natural; bowels regular.

℞. Tr. Kreosote ʒiv.

Aquæ ℥vi.

Apply to gums as before.

*8th.*—No improvement since last note; ulceration has gradually extended; the roots of the lower incisors are somewhat exposed by the removal of the gums by ulceration; anorexia. The ulcerated surface was now touched with the nitrate of silver. Wash continued.

*12th.*—The ulceration has not extended laterally since the 8th; the alveoli of the lower incisors are exposed, and slight caries has taken place. The use of the nitrate of silver has been continued, and the following wash was applied:

℞. Sulph. Cupri ʒii.

Pulv. Cinchonæ ℥ss.

Aquæ ℥iv.

To be carefully applied three times a day.

*14th.*—Ulceration not much improved. The exterior of the chin has assumed a leaden colour; a vesicle has formed on the chin of a purple cast. A common cataplasm was applied. Treatment continued.

*16th.*—The vesicle bursted on 15th, and to-day was followed by a slough one-fourth of an inch in diameter; anorexia continues; patient quite feeble, somewhat emaciated. Half a grain of quinia was given every two hours. Ordered a cataplasm composed of pulv. cinchon. rhub., and flaxseed meal to chin.

*17th.*—Slough increased in size; it is a half inch in diameter; patient very feeble, no appetite. In addition to the quinia, beef-essence and wine-whey were given. Fœtor intolerable, to correct which a solution of kreosote was applied.

*20th.*—The ulcer measures one inch and a quarter laterally, and one inch perpendicularly; it includes nearly the whole chin; the bone is exposed; the ulcer looks more healthy; patient continues very feeble. Quinia, beef-essence, and wine-whey continued. The following local application was now made: ℞. Vin Aromaticum; lint was saturated with this, and applied. Tr. opii gtt. x. nocte. On the following day, a poultice composed of

℞ Pulv. Cinchon. Rub.

Fœces Opii āā ʒi.

Farin. Lini ℥ii.

was applied over the lint saturated with aromatic wine.

*24th.*—Ulcer has not extended since the date of last note; it presents a more healthy appearance; but little change in the aspect of the exposed bone. Some difficulty in taking the quinia by the mouth; therefore, quiniæ gr. ii., amyli ʒi. was given by injection every three hours. Tr. Mur. Ferri was applied to the ulcer, as we were not able to obtain more of the aromatic wine.

*26th.*—Ulcer perfectly clean, and appears disposed to heal at one edge; some healthy granulations are apparent; dry lint was now applied. Quinia injections continued.

*30th.*—Considerable improvement in appearance of ulcer; cicatrization has commenced; about one inch of the inferior maxillary bone is still exposed; strength of patient increased; appetite improving; slight attack of diarrhœa. Ordered Tr. opii gtts. vii., amyli ʒi., by injection every three hours. Treatment continued.

*April 2d.*—Diarrhœa arrested; healthy granulation has commenced, and the ulcer is rapidly cicatrizing. Strength much improved; appetite good; local application continued; quiniæ gr. ½ every three hours; diet of the most nutritious character.

*9th.*—The patient has gradually improved up to this date; the ulcer is healing slowly. A few days past an ulcer was discovered on the inner side of right cheek, which has perforated the bone near the angle of the jaw; this was arrested by the nitrate of silver. A strong solution of iodine was applied to the exposed bone, which application was continued until the bone exfoliated; the ulcer then healed, leaving a very unseemly cicatrix.

*General Summary of the Treatment pursued.*

When the disease first appeared, the mineral acids, variously diluted, were used, but with little advantage. We also tried the sulphate of zinc, but the result of this remedy was equally unsatisfactory. At the suggestion of Professor Dunglison, kreosote was used, but this article had a very limited effect.

The treatment which we found successful in the greatest number of cases, was the following: In the commencement of the disease, when the gums presented the white, spongy appearance, already described, accompanied with a partial separation of the gums from the teeth, we resorted to the following application:

℞ Sulph. Cupri ʒi—ii.

Pulv. Cinchonæ ℥ss.

Aquæ ℥vi.

This was carefully applied to every part of the mouth that presented the appearance of disease, three times a-day. If, by this means, the progress of the disease was not arrested, in addition to the treatment above mentioned, we found the nitrate of silver productive of much benefit, when applied after the commencement of ulceration. By its use we could, in a measure, destroy the diseased portions, and often excite a new and healthy action in the part affected, and in this manner prevent the extension of the ulceration. In the case reported, by the advice of Dr. Gerhard, the aromatic wine was used, also the muriated tincture of iron, with the happiest effect, after a portion of the chin had sloughed away, leaving the bone exposed. When the cheek became turmid and hard, leeches and blisters were applied; but they were productive of but very little, if any, advantage. After the cheek, or part affected, assumed the purple colour described, followed by the formation of vesicles, a common poultice was used; but when sloughing commenced, the fermenting poultice was substituted until the gangrenous sloughs were arrested. To correct the fœtor, which was often very great, the pyroligneous acid, or a solution of the chloride of soda, was used. At this stage of the disease, we found it necessary to administer tonics, conjoined with the most nutritious diet; quinia was the tonic to which we gave preference. When diarrhœa supervened, some mild aperient was given to carry off all irritating matter contained in the intestines; after which, some astringent was administered. As diarrhœa almost uniformly comes on towards the close of this disease, it may depend on the putrid matter which is discharged from the gangrenous surface, finding its way into the stomach. Almost every child which suffered from gangrenous sore mouth, was at the time convalescing from rubeola; in many cases, therefore, we found it necessary to give tonics from the commencement of the attack. The diet of these children was of the most nutritious character, as eggs, beef-essence, &c.; the rooms in which they were placed, were large and airy; if the strength of the patients would permit it, they were taken by their nurses in the open air every morning and evening. When it was possible to do this, it was found very beneficial. Of the twenty-three cases treated for gangrenous sore mouth, nineteen recovered, and four proved fatal. This disease, which was here a sequela of *rubeola*, proved to be the most disagreeable and distressing in its character, of any of the sequelæ that occurred during the epidemic; but pneumonia proved far more fatal.

[The gangrenous sore mouth is a disease with which every practitioner should be acquainted, as it not unfrequently occurs in debilitated children, more especially, as already stated, at the termination of measles. Its effects so nearly resemble those of mercury, when pushed too far, or administered in a case where there is some peculiar idiosyncrasy, that practitioners are oftentimes unjustly censured on this account, from a supposed injurious consequence of the mercurials which they may have prescribed. Still, I have no doubt that mercury acts in the same direction, if we may use the term, as gangrenous sore mouth, and tends to increase its mischievous effects, just as it does of venereal phagedœna.

I have seen the same disease in adults, and then it was usually complicated with gangrene of some other organ, especially of the lungs. The same complication often occurs in children, showing that there is a general vitiation of the fluids, which is quite as important as the local mischief.

Of the treatment, as laid down by Dr. Webb, we entirely approve. The experience, both at the Children's Hospital at Paris and Philadelphia, as well as that, I believe, of other institutions, is perfectly uniform. The general system should be supported by tonics, and the local mischief should be arrested by remedies which act as powerful modifiers of the tissues adjoining the diseased part, and not yet passed into sphacelus. Hence, caustics which possess the double advantage of destroying the part which is already dead matter, and of acting on the surrounding tissues, are generally the most appropriate. At Paris we always used the nitrate of silver; in this country, this invaluable remedy is used, as well as strong solutions of sulphate of copper. Either caustic is much more readily and completely applied in the form of a strong solution, (say 20 grains to the ounce,) than in substance, for it may be made to include the diseased tissue much more completely.

I suggested, in one case, the use of the muriated tincture of iron, without dilution, as a caustic, and after dilution, as a simple stimulant, from observing its good effects in a case of venereal phagedœna. The aromatic wine of the French pharmacopœia is a remedy which was introduced lately into tolerably general use at Philadelphia, particularly from the excellent results obtained by Dr. Ricord, of Paris, which were witnessed by many of our countrymen. It is an invaluable remedy, not only in venereal ulcers, but in sloughing sores in general, stimulating powerfully the adjacent tissues, and giving new activity to the tissues which are infiltrated with serum, previously to falling into gangrene.

The aromatic wine is readily prepared; we will give the formula in another number.

W. W. G.]

---

*Cincinnati College.*—Dr. Gross has resigned the Chair of General Pathological Anatomy, in the Medical Department of the Cincinnati College.

